# Nurse-midwives’ ability to diagnose acute third- and fourth-degree obstetric lacerations in western Kenya

**DOI:** 10.1186/s12884-017-1484-4

**Published:** 2017-09-18

**Authors:** Leeya F. Pinder, Kelsey H. Natsuhara, Thomas F. Burke, Svjetlana Lozo, Monica Oguttu, Leah Miller, Brett D. Nelson, Melody J. Eckardt

**Affiliations:** 1 Division of Global Health and Human Rights, Department of Emergency Medicine, Massachusetts General Hospital, 125 Nashua Street, Suite 910, Boston, MA 02114 USA; 2 Harvard T.H. Chan School of Public Health, Boston, MA USA; 3 Harvard Medical School, Boston, MA USA; 4 Kenyan Medical and Educational Trust (KMET), Kisumu, Kenya

**Keywords:** Obstetric laceration, Obstetric tear, Obstetric fistula, Third- and fourth-degree perineal laceration, Obstetric anal sphincter injuries (OASIS), Maternal health, Obstructed labor, Developing countries, Kenya

## Abstract

**Background:**

Obstetric fistula devastates the lives of women and is found most commonly among the poor in resource-limited settings. Unrepaired third- and fourth-degree perineal lacerations have been shown to be the source of approximately one-third of the fistula burden in fistula camps in Kenya. In this study, we assessed potential barriers to accurate identification by Kenyan nurse-midwives of these complex perineal lacerations in postpartum women.

**Methods:**

Nurse-midwife trainers from each of the seven sub-counties of Siaya County, Kenya were assessed in their ability to accurately identify obstetric lacerations and anatomical structures of the perineum, using a pictorial assessment tool. Referral pathways, follow-up mechanisms, and barriers to assessing obstetric lacerations were evaluated.

**Results:**

Twenty-two nurse-midwife trainers were assessed. Four of the 22 (18.2%) reported ever receiving formal training on evaluating third- and fourth-degree obstetric lacerations, and 20 of 22 (91%) reported health-system challenges to adequately completing their examination of the perineum at delivery. Twenty-one percent of third- and fourth-degree obstetric lacerations in the pictorial assessment were incorrectly identified as first- or second-degree lacerations.

**Conclusion:**

County nurse-midwife trainers in Siaya, Kenya, experience inadequate training, equipment, staffing, time, and knowledge as barriers to adequate diagnosis and repair of third- and fourth-degree perineal tears.

## Synopsis

Nurse-midwives in western Kenya have multiple barriers to diagnosing third- and fourth-degree obstetric lacerations, a finding which may contribute to the overall fistula burden in Kenya.

## Background

Obstetric fistula (OF) is a disabling medical condition that results in fecal and/or urinary incontinence due to the breakdown of tissue separating the vagina from the bladder and/or rectum [1]. OF affects approximately two million women globally, with an estimated 50,000–100,000 newly affected women annually [2]. Women affected by OF often face extreme marginalization and discrimination [3].

OF is a complication of childbirth that has been nearly eliminated in high-income countries, but continues to burden women in low- and middle-income countries [1, 4]. In these settings, obstructed labor is thought to account for the majority of OF cases [5, 6]. In a recent study, our team discovered that 31.7% of women undergoing surgical repair for OF at two large Kenyan fistula camps actually suffered from unrepaired third- and fourth-degree obstetric perineal lacerations rather than true OF [7]. Third- and fourth-degree perineal lacerations, also known as obstetric anal sphincter injuries (OASIS), are characterized by tears from the vagina into and through the anal sphincter, respectively, and often result in symptoms indistinguishable from the more traditionally described OF (i.e., uncontrolled loss of flatus and stool) [8]. These anal sphincter injuries are not caused by obstructed labor but from trauma during passage of the baby upon exiting the vagina.

In high-income countries, OASIS are identified at the time of delivery and are primarily repaired. In contrast, there is very little understanding about skilled birth attendant competencies for identifying and either repairing or referring these injuries in low- and middle-income countries. The World Health Organization (WHO) defines skilled birth attendants as “an accredited health professional such as a midwife, doctor or nurse who has been educated and trained to proficiency in the skills needed to manage normal (uncomplicated) pregnancies, childbirth and the immediate postnatal period, and in identification, management and referral of complications in women and newborns” [9]. A skilled birth attendant is present in approximately 44% of all deliveries in Kenya [10], the majority of whom are nurses/midwives (64%) [11]. In this study, we hypothesized that barriers to timely repair of third- and fourth-degree perineal tears in western Kenya included failure to diagnose due to knowledge gaps and health system dysfunction. We, therefore, conducted a multi-method assessment of post-delivery practices and competencies of one cadre of skilled birth attendants, Kenyan nurse-midwives, with the hope that the results could inform the development of an innovative community-based solution.

## Methods

A written survey and a pictorial assessment tool were developed by U.S. obstetricians/gynecologists and reviewed with Kenyan Family and Emergency Medicine postgraduates (residents) and a Kenyan clinical officer for cultural context, clarity of questions, and feasibility of design. Adjustments were made as recommended. The Siaya County Ministry of Health Maternal Health Taskforce directed that the assessment take place during a previously planned one-day county maternal health-training program for nurse-midwife “Maternal Health Trainers” from each of the seven Siaya sub-counties. Maternal Health Trainers were appointed by their respective counties based on experience, skills, and desire to serve as maternal health advocates for their county. Verbal informed consent was obtained from each participant prior to administration of the assessment.

The final assessment tool contained color images (used with permission from the Perineal and Anal Sphincter Trauma Program, Croydon University Hospital, Croydon UK) of normal and abnormal perineal anatomy (e.g., obstetric lacerations) with accompanying clinical scenarios. During the assessment, images (some illustrative diagrams and some actual images of perineal tears) were sequentially projected on a screen as well as on tablets/computers, which allowed for high-resolution views. Research staff gave clinical scenarios verbally. Each participant marked their own responses on separate scorecards, and the group was proctored to be certain that answers were individual. Some images required identification of perineal anatomy (e.g., anal sphincter, rectal/anal mucosa, and muscles of the perineal body) and others required diagnosis of the degree of laceration (included ten pictures: one of a first-degree tear, one of a button-hole tear, and eight of third- or fourth-degree tears). Answers were recorded as correct if the participant correctly identified the degree of laceration or identified an OASIS and/or stated the general structure (e.g., sphincter, anus, muscle, etc.), but a correct answer did not require the formal anatomical name. Study personnel were available to provide clarification of any questions. An accompanying open-response survey collected information on participant demographics, training and experience with obstetric tears, and barriers to adequate perineal laceration diagnosis and repair.

De-identified demographic data and the assessment and survey responses were entered into an encrypted database. Frequency analyses were performed for each of the survey questions using Microsoft Excel 2015 (Seattle, Washington, U.S.).

Study approval was obtained from the Siaya County Ministry of Health (Siaya, Kenya) and the institutional review board of Partners HealthCare (Massachusetts General Hospital, Boston, MA, USA).

## Results

Twenty-two of the 22 (100%) nurse-midwife Maternal Health Trainers, from the seven sub-counties of Siaya County who attended a one-day maternal health-training program in September of 2015, agreed to participate in the assessment. Twelve (54.5%) of the 22 Maternal Health Trainers were female, the mean years of post-graduate clinical experience was 16.0 (range 1–28), and the mean number of deliveries attended per Maternal Health Trainer in the month immediately prior to the survey was 17.6 (range 0–77) (Table 1). Six participants did not respond to the question regarding post-training experience.

**Table 1:** Participant Demographics

Maternal Health Trainers and their sub-counties of origin	
Alego Usonga	2 (9.1%)
Bondo	6 (27.3%)
Gem	4 (18.2%)
Rarieda	1 (4.5%)
Siaya	3 (13.6%)
Ugenya	2 (9.1%)
Ugunja	4 (18.2%)
Gender (n = 22)	
Female	12 (54.5%)
Male	10 (45.5%)
Mean years of post-training experience (n = 22)	16.0 (± 9.0)
< 5 years	1
5–9 years	4
> 10 years	11
No response	6
Deliveries performed in the prior month (*n* = 21)	
Collective total	369
Mean per person	24.6 ± 22.5
Mean number of tears repaired in the prior month (*n* = 20)	
First- or second-degree repairs	2.2 ± 3.3
Third- or fourth-degree repairs	0.05 ± 0.2

Fourteen (63.6%) Maternal Health Trainers reported having received training in visual and manual evaluation to identify first- and second-degree obstetric lacerations, while only four of the 22 (18.2%) had received training on evaluating for third- and fourth-degree obstetric lacerations. Two of the 22 (9.1%) respondents reported routinely performing rectal exams to evaluate the perineum following delivery (Table 2). None of the participants felt comfortable with repairing OASIS.

**Table 2:** Participants’ experience with third- and fourth-degree obstetric lacerations

	Prevalence
Routinely perform rectal exams on all patients after delivery (*n* = 20)	2 (10.0%)
Perceive a good understanding of the muscles that can be damaged during delivery (*n* = 21)	11 (52.4%)
Previous training in perineal tear evaluation (*n* = 22)	14 (63.6%)
Previous training specifically in third- and fourth-degree tear evaluation (*n* = 22)	4 (18.2%)
Comfortable repairing a third- or fourth-degree tear (n = 22)	0 (0.0%)
Reported evaluation of tears after delivery was challenging (*n* = 22)	20 (90.9%)
Specific challenges to perineal evaluation following delivery (Open Response)	(n = 22)
Staff shortage / workload	13 (59.1%)
Inadequate or broken supplies	12 (54.5%)
Deliveries occurring at night	9 (40.9%)
Inadequate light source	8 (36.4%)
Lack of pain control	1 (4.5%)
Distance in km to nearest referral center for complex repair (excluding providers based at a referral center) (*n* = 16)	25.3 ± 10.8
Distance ≤ 10 km	2
Distance 10-20 km	2
Distance >20 km	11
No response	1
Participants with system to follow-up on patients who have been referred for repair (n = 20)	12 (60.0%)

Fifteen of 22 (68.2%) respondents described that the mean distance to a referral center where an obstetric laceration repair could be performed was 25.3 (range 10–50) kilometers. Twelve of 20 (60%) respondents reported having a system for follow-up after referral for OASIS repair (Table 2). Twenty of 22 participants (90.9%) reported other health systems barriers to perineal tear diagnosis; under-staffing, heavy workloads, inadequate supplies, deliveries occurring at night, and inadequate light sources all contributed to making evaluation of the perineum difficult following delivery (Table 3).

**Table 3:** Participant scores on pictorial laceration assessment

Skill assessed (*n* = 22)	Mean percent of pictorial questions answered correctly (standard deviation)
Classification of laceration as either simple (first/second-degree) or complex (third/fourth-degree) (9 images)	76.1 ± 15.3
Identification of laceration as third/fourth- degree (8 images.)	69.9 ± 17.5
Identification of structures of the anal sphincter complex (5 images)	26.4 ± 25.7
Recognition of the anus or rectal mucosa when it is torn (2 images)	25.0 ± 25.6
Identification of the sphincter muscle (not specific to internal or external) (3 images)	21.2 ± 28.3
Overall anatomical structure identification (10 images)	16.4 ± 16.0

The 22 Maternal Health Trainers correctly identified third- or fourth- degree obstetric lacerations in a mean of 5.6 out of eight (69.9%) OASIS images presented in the pictorial assessment. A mean of 1.6 of five (26.4%) depictions of the anus and anal sphincter complex were correctly identified and only a mean of 0.5 of five (10.9%) muscles of the perineum were identified correctly in the assessment. (Table 3).

## Discussion

Obstetric fistula and unrepaired OASIS have profound social implications. Symptoms of incontinence associated with OF result in women often being ostracized by their communities and abandoned by their husbands and families [12]. Without treatment, women affected by OF have few prospects for work, and their isolation may lead to depression and even suicide [4, 6]. In our previous study on OF, almost one-third (31.7%) of women studied had unrepaired OASIS rather than a true OF [7]. Immediate OASIS repair, therefore, can avoid these symptoms for approximately a third of women otherwise destined to encounter these consequences.

This study identified several barriers that would need to be addressed for improved acute OASIS diagnosis and subsequent repair. The delivery provider in low- and middle-income countries is usually a mid-level provider, such as a nurse-midwife, and plays the critical role of having to identify sphincter injuries. However, only 4 (18.2%) of the midwife trainers in this study had received training in identification of these injuries. Heavy workloads, lack of supplies, and poor lighting at night were reported as specific challenges to perineal evaluation. If complex tears were found, midwives were not trained and lacked the specific knowledge and skills to repair these injuries. Usually a clinician with the skills necessary to repair the laceration was not on site and transportation needed to be arranged to the nearest sub-district, district, or county referral hospital hoping that a provider skilled in repair would be available – a mean distance in this study of more than 25 km. Training programs for midwife identification and management of complex perineal tears are imperative, but they must be designed along with system interventions that address these issues.

This study found that identification of OASIS on diagrams and images by experienced nurse-midwives who serve as maternal health trainers was poor, and it is suspected that knowledge among non-maternal health trainers may be even more limited. While the correlation is unclear between being able to identify obstetric lacerations on two-dimensional images versus being able to identify OASIS after delivery, the inability to demonstrate essential knowledge of anatomy and identification of tear severity, along with provider admission of poor training and failure to routinely examine patients, suggest that most acute third- and fourth-degree obstetric lacerations may be incorrectly identified as minor lacerations thereby missing the opportunity for proper repair immediately following delivery.

Several studies have shown, following a structured evidence-based training program in identifying and repairing OASIS, participants classified OASIS more accurately [13–16]. In a study by Andrew et al. evaluating an OASIS repair training program in the United Kingdom, there was a significant change in provider ability to identify and repair the internal sphincter following training (60% vs. 90%; *P* < 0.0001) [13]. Still needed is the development of a best-practice training model for improving the identification and management of acute OASIS among mid-level providers in a resource-constrained environment.

When considering the difficulties of referral and transport, coupled with the low numbers of physicians and medical officers in settings such as western Kenya, task-shifting and equipping nurse-midwives with the necessary skills to repair OASIS at the community level becomes an important consideration. This would necessitate additional research and innovative policy changes to support task shifting, training, and empowering nurse-midwives to repair these injuries at the time of primary identification.

One of the limitations of this study is the relatively small sample size and non-randomized methodology. However, this cohort had representatives from each of the sub-counties in the region and was chosen in accordance with the Ministry of Health’s selection for Maternal Health Trainers including experience, knowledge, and skills. While we utilized a representative sampling of nurse-midwives from the region, the practices described may not be generalizable to other regions of Kenya or to sub-Saharan Africa, and additional studies may be needed. A portion of the survey included self-reported data, which are subject to recall and social desirability biases. Additionally, this study utilized an obstetric laceration interactive pictorial tool to assess the knowledge of the participants. While the pictures clearly depicted obstetric lacerations, this assessment tool has not been fully validated in this setting and did not include a photograph of a second-degree laceration. Further, it is unclear whether the ability to recognize perineal tear severity and anatomy on two-dimensional images correlates well with the three-dimensional evaluation of actual damaged tissue of the perineum. The assessment was designed to be practical, feasible, and acceptable through utilization of images from an existing evidence-based training program and through iterative refining and vetting by independent developed-country and local clinicians. Additional assessments on three-dimensional tissue models or on postpartum patients may be more representative, but the low test scores, considered in concert with the reported lack of training and lack of a regular, thorough perineal examination of patients, support the assertion that poor diagnosis of OASIS is a significant concern in the prevention of OF in this setting.

## Conclusion

Nurse-midwives in western Kenya lack knowledge, training and skills in perineal evaluation after birth. Failure to diagnose is an important barrier to timely repair. This coupled with poor lighting, understaffing, inadequate supplies, and inadequate referral systems to distant facilities all present significant challenges to accurate identification and repair of third- and fourth-degree obstetric lacerations and may result in the symptoms and social consequences of incontinence and increase the global burden of obstetric fistula. To reduce the burden of unrepaired third- and fourth-degree obstetric lacerations, the creation of a best-evidence training package is necessary to address the gap in ability, skills, and resources for appropriate diagnosis and repair of OASIS. Further research, strengthening of referrals, collaboration with policymakers in consideration of task shifting, and advocacy from maternal health proponents is needed to eliminate the burden of this maternal complication.

## Abbreviations

OASIS: obstetric anal sphincter injury
OF: obstetric fistula
